# Functional and radiographic outcomes of allograft reconstruction for contained glenoid defects in revision reverse total shoulder arthroplasty: a case series

**DOI:** 10.1016/j.jsea.2026.100054

**Published:** 2026-06-26

**Authors:** Kyle B. Christy, Maarouf A. Saad, Randall A. Arroyo, Christopher D. Joyce, Peter N. Chalmers, Robert Z. Tashjian

**Affiliations:** a Department of Orthopaedics, University of Utah, Salt Lake City, UT, USA; b School of Medicine, University of California, Riverside, CA, USA

**Keywords:** Bone grafting, Shoulder, Arthroplasty, Revision shoulder arthroplasty, Reverse total shoulder arthroplasty, Allograft, Glenoid defect

## Abstract

**Background:**

Glenoid bone defects are often encountered in revision shoulder arthroplasty and in the setting of revision reverse total shoulder arthroplasty (rTSA); allograft reconstruction is a commonly used technique to manage these defects. Prior studies have reported outcomes after glenoid bone grafting during revision rTSA; however, there is limited evidence on the outcomes following glenoid bone grafting for contained, central glenoid defects during revision rTSA. The purpose of this study was to evaluate the short-term clinical and radiographic outcomes following revision rTSA with glenoid bone grafting for a contained central glenoid defect using femoral head allograft.

**Methods:**

In this retrospective case series, patients who underwent glenoid allograft reconstruction during revision rTSA for contained glenoid defects with a minimum 2-year follow-up were included. Intraoperatively, a femoral head allograft was shaped to match a cement mold of the defect and impacted into the central defect followed by placement of a long-post baseplate. Beta angle and Nerot-Sirveaux Classification (if applicable) were assessed on pre-operative radiographs. On post-operative radiographs, beta angle, Nerot-Sirveaux Classification, implant shifting, gross loosening, and graft incorporation were assessed. Active forward elevation was measured pre-operatively and post-operatively. At 2-year follow-up, visual analog scale pain scores and American Shoulder and Elbow Surgeons scores were collected.

**Results:**

Thirteen patients underwent glenoid allograft reconstruction during revision rTSA for a contained glenoid defect by a single surgeon during the study period. Eleven of 13 patients had radiographic and clinical follow-up (47 ± 22 months, range: 24-82 months), with a beta angle of 83.2° ± 11.3° pre-operatively and 89.1° ± 12.0° post-operatively (*P* = .156). On the 2 patients revised from an rTSA, Nerot-Sirveaux classifications were 0 and 3, and post-operatively patients had a Nerot-Sirveaux classification of 1 ± 1 (none: 3, grade 1: 5, 2: 1, 3: 2). All patients had radiographic evidence of graft incorporation. At final post-operative follow-up, visual analog scale pain scores were 1.1 ± 1.4 and American Shoulder and Elbow Surgeons scores were 80 ± 14. At 25 months post-operatively, one patient was revised due to a recurrent infection.

**Conclusion:**

In the short to mid-term, revision rTSA with allograft reconstruction for contained central glenoid defects using a long-post baseplate provides excellent radiographic healing, improved range of motion, good function, low pain, and low baseplate revision rates. This technique may represent an effective option for managing contained central glenoid defects in the setting of revision rTSA.

The incidence of revision shoulder arthroplasty in the United States has increased 153% from 2012 to 2018,^3^ which coincided with an increase in the incidence primary total shoulder arthroplasty from 16.8 to 31.8 per 100,000 people in the same time period.^2^ Reverse total shoulder arthroplasty (rTSA) is the most common revision operation performed.^3^^,^^8^^,^^17^ Glenoid defects are often encountered in revision rTSA and can be managed intraoperatively using augmented components, bone grafting, or a combination of these.^4^ Current standard augmented baseplates are designed with limited sizes, and companies may not offer an implant with adequate augment thickness to address the many defects.^15^ Although custom, patient-specific augmented baseplates can be used, they are expensive and take up to 12 weeks to manufacture, during which the underlying glenoid morphology can continue to change, potentially limiting the component's fit. This has made bone grafting a commonly employed strategy in correcting glenoid defects.^18^

The current literature on the success of bone grafting, specifically the use of an allograft, to treat glenoid defects during rTSA is conflicting. In patients undergoing structural glenoid allograft during rTSA, the reported complete graft incorporation rate ranges from as high as 82% to as low as 30% at a minimum 1-year follow-up.^11^^,^^16^^,^^19^ Specifically in patients undergoing revision rTSA with concurrent glenoid grafting, outcomes are also varied. After a failed anatomic total shoulder arthroplasty (aTSA), Melis et al used a combination of structural iliac crest autograft, cancellous autograft, and structural allograft and reported a graft healing rate of 76% at a minimum of 2-year follow-up (average 47 months).^11^ Kusin et al reported on femoral head allograft reconstruction of the glenoid in the setting of failed aTSA, rTSA, or hemiarthroplasty and demonstrated an 86% graft healing rate at a minimum of 2-year follow-up.^9^^,^^14^ These prior publications did not differentiate between contained glenoid defects where all glenoid walls were intact vs. uncontained defects where at least one glenoid vault wall was insufficient. We previously reported the outcomes of large bulk allograft reconstructions for uncontained glenoid defects in the setting of revision rTSA and reported a failure rate of 41.2% at minimum 5-year follow-up.^7^ Currently there are limited data regarding the structural and functional outcomes following allograft reconstruction of contained glenoid defects in the setting of revision rTSA.

Therefore, the purpose of this study was to evaluate the short-term to mid-term clinical and radiographic outcomes following revision rTSA with allograft reconstruction for a contained glenoid defect. We hypothesize that the use of an allograft for contained glenoid defects during revision rTSA would result in high rates of graft incorporation, low rates of revision, and excellent patient-reported outcomes (PROs).

## Methods

### Study population

**Figure 1:**
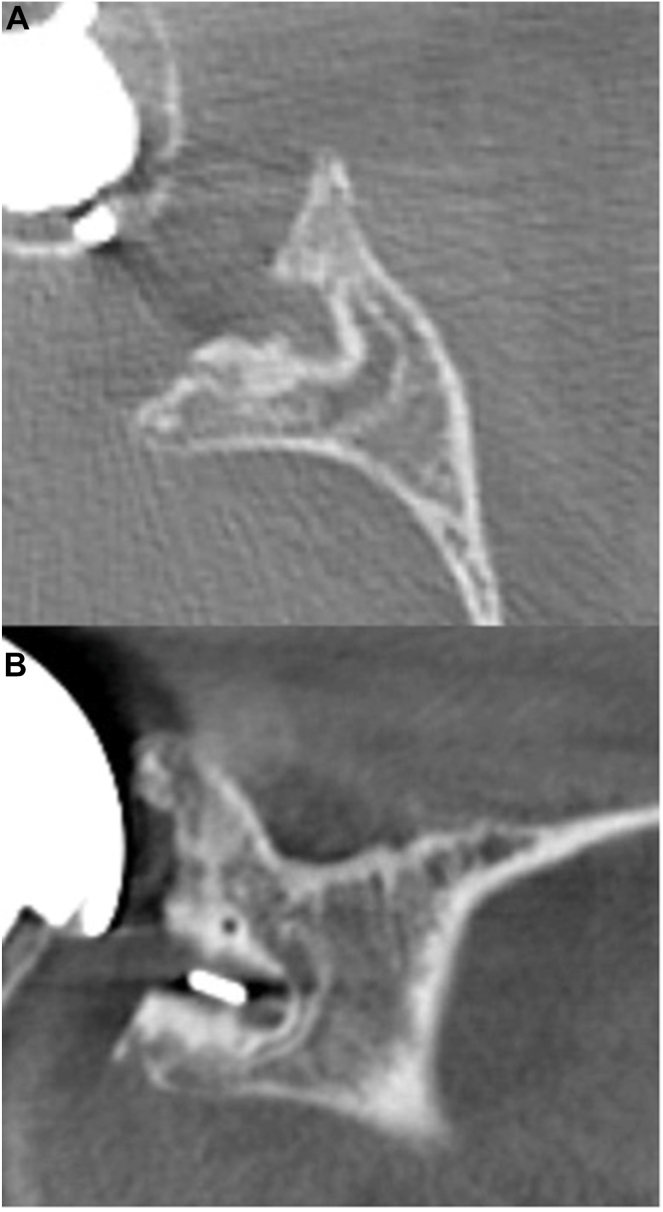
Two-dimesional computed tomography (CT) images of the contained glenoid defect. (**A**) Axial and (**B**) coronal images.

This is a retrospective consecutive case series in an academic setting conducted with institutional review board approval. The operative log of a fellowship-trained shoulder and elbow surgeon (R.T.) from 2015-2023 was reviewed. Patients with a minimum 2-year follow-up who underwent revision rTSA with concominant glenoid bone grafting with a femoral head allograft for contained, central glenoid defects were included. The presence of a contained glenoid defect (intact vault walls with a central vault defect only) was confirmed using pre-operative computed tomography (CT) scans (Fig. 1).

The surgeon made the decision to perform a revision to rTSA with a structural allograft based on whether baseplate inclination could be corrected to at least neutral on a standing true anteroposterior (Grashey) radiograph and version to within 10° of neutral on an axillary radiograph, without requiring reaming of more than 5-10 mm of glenoid bone stock. The objectives of reaming were to achieve 100% baseplate seating and restore the joint line to its native position. If these goals could not be met with reaming alone, rTSA with structural allografting was performed. Structural allografting was not used when lateralization of the joint line was the sole objective and adequate correction could be achieved within the previously mentioned reaming limits. No revision rTSA with glenoid graft reconstruction was performed using autograft during the study period.

### Surgical technique

A standard deltopectoral approach was used. Both the Aequalis Reversed Shoulder Arthroplasty System (Tornier, Bloomington, MN, USA) (15 patients) and the Perform Reversed Augmented Glenoid (Tornier) (1 patient) were used. First, the humeral head and stem were removed, and the remaining humeral shaft prepared per manufacturer's recommendations. The glenoid components were subsequently exposed and if not completely loose, were removed with osteotomes and the instruments associated with the implantation. At this point, the glenoid was assessed, and all excess cement was removed. Then the glenoid cavity was curetted and burred to bleeding bone. Next, a small batch of cement was mixed and used to create a mold of the glenoid defect. A femoral head structural allograft was hand-shaped to the defect using the mold and a burr and the graft was impacted into the central defect and held peripherally with wires (Video S1).

**Figure 2:**
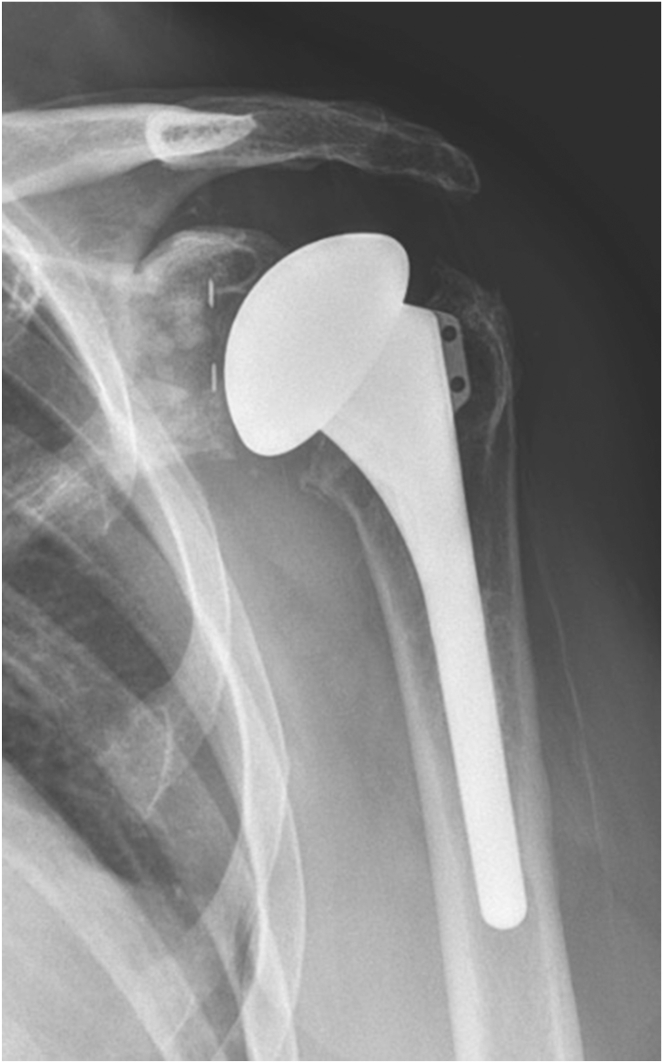
Pre-operative true anteroposterior radiograph of the shoulder with a failed anatomic total shoulder arthroplasty with a loose glenoid component.

**Figure 3:**
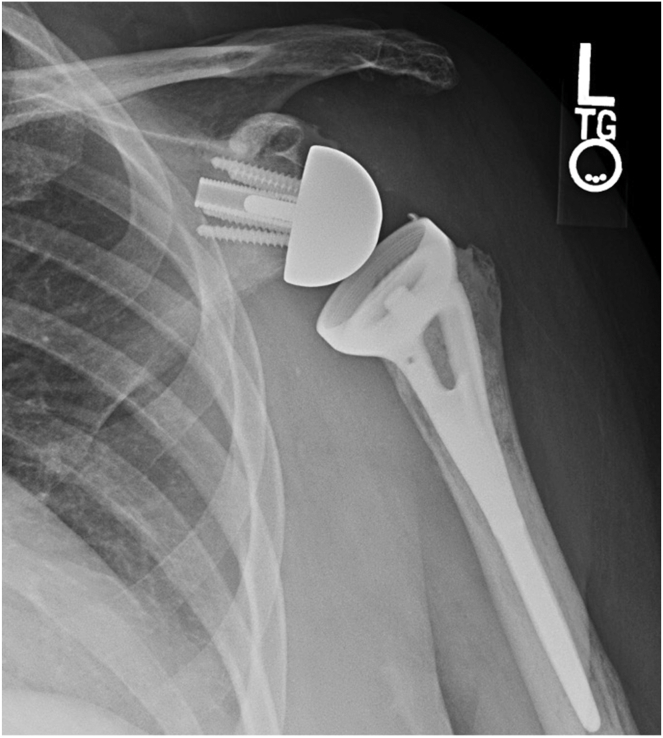
Post-operative true anteroposterior radiograph of the shoulder after rTSA with an allograft reconstruction of a central glenoid defect. *rTSA*, reverse total shoulder arthroplasty.

After graft impaction, a central guide pin was placed so that retroversion was reduced to less than 10° and superior inclination to 0° based on the floor of the supraspinatus fossa. Once the graft and guide pin were in place, the glenoid was prepped for the baseplate per the manufacturer's recommendations. A 25-mm long post was used in all cases where the Aequalis Reversed Shoulder Arthroplasty System was used to engage in at least 5 mm of native glenoid. The baseplate was subsequently impacted into place, and the screws placed through the baseplate and graft and into the native glenoid. First, the nonlocking screws (2 in total) were placed to compress graft and then the locking screws were placed (2 in total). In the patient who had a Perform Reversed Augmented Glenoid, a 6.5-mm central screw was used to engage the far cortex of the glenoid vault. At this point, the remainder of the rTSA was performed. A standard Grammont-style humeral stem (155° neck shaft angle) was used in all cases with the stem placed between 0° and 20° of retroversion (Video S1). No biologic augmentations were used in any cases (Figs. 2 and 3).

The post-operative rehabilitation protocol included a previously described home exercise program.^5^ After 3 months post-operatively, patients were prescribed in-person physiotherapy for 1-2 days per week in addition to their home exercise program.

### Clinical data collection

For each patient, the following data were collected: demographics, surgical details, post-operative complications, radiographic measurements, active forward elevation (AFE), visual analog scale (VAS) pain score (0, no pain at all; 10, severe pain), and American Shoulder and Elbow Surgeons (ASES) score.

### Radiographic evaluation

Pre-operative and minimum 2-year post-operative radiographs were independently evaluated by 2 authors (K.C. and M.S.). Beta angle was measured on Grashey views pre-operatively and post-operatively as previously described.^13^^,^^21^ For patients with a prior failed rTSA, the Nerot-Sirveaux classification was used to grade scapular notching on pre-operative radiographs.^22^ On final follow-up radiographs, scapular notching was graded using the Nerot-Sirveaux classification, the allograft was evaluated for incorporation (complete, partial, or none), and the baseplate was evaluated for loosening and migration.

### Statistical analysis

Inter-rater reliability was assessed using the intraclass correlation coefficients using a 2-way random effects, average measurement, absolute agreement model and subsequently evaluated as: ≤0.400, poor; 0.401 to 0.600, fair; 0.601 to 0.750, good; and 0.751 to 1.000, excellent.^6^ Pre-operative and post-operative beta angle, AFE, and PROs were compared using a paired *t*-test. Statistical significance was defined as *P* ≤ .050. All analyses were performed in SPSS (v30, IBM, Armonk, NY, USA).

## Results

From 2015-2023, 16 patients underwent revision rTSA with allograft reconstruction for a contained glenoid defect. Three patients were excluded due to infection with revision to an antibiotic spacer <2 years post-operatively, leaving 13 patients (6 males, 7 females; age at surgery: 51.8 ± 7.3 years) available for follow-up. American Society of Anesthesiologists scores and smoking status at the time of revision rTSA are outlined in Table I. The indications for revision rTSA were as follows: 5 patients (39%) for rotator cuff tearing after aTSA, 3 patients (23%) for glenoid component loosening after aTSA, 2 patients (15%) for both rotator cuff tearing and glenoid component loosening after aTSA, 2 patients (15%) for baseplate loosening after rTSA, and 1 patient (8%) for continued pain and dysfunction and failing conservative management after aTSA. 11 of 13 (85%) patients were revised from an aTSA, and 2 of 13 (15%) were revised from an rTSA.

**Table I:** Patient demographics

Variable	
ASA score	2.5 ± 0.5
Smoking status	
Current	1 (8%)
Former	4 (31%)
Never	8 (62%)

*ASA*, American Society of Anesthesiologists.

Patient American Society of Anesthesiologists (ASA) score and smoking status at the time of surgery. ASA score is presented as average ± SD; smoking status is presented as n (%).

Intraclass correlation coefficients for all measurements were rated as excellent (≥0.861). Eleven of 13 (85%) patients had radiographic follow-up (47 ± 22 months; range: 24-82 months). Beta angle was measured at 83.2° ± 11.3° pre-operatively and 89.1° ± 12.0° post-operatively (*P* = .156) (Table II). Pre-operatively, 1 of 2 patients revised from an rTSA had no scapular notching and the other had grade 3 scapular notching according to the Nerot-Sirveaux classification. Post-operatively, 3 patients (27%) had no scapular notching, 5 (45%) had grade 1, 1 (9%) had grade 2, and 2 (18%) had grade 3 scapular notching (Table I). In all 11 patients, there was radiographic evidence of complete graft incorporation. One patient had radiographic evidence of a baseplate shift in position, although this stabilized and the graft was healed. Eleven of 13 (85%) patients had PROs collected at 49 ± 19 months (range: 27-82 months).

**Table II:** Radiographic and functional outcomes

Variable	
Beta angle	
Pre-operative	83.2° ± 11.3°
Post-operative	89.1° ± 12.0°
Active forward elevation∗	
Pre-operative	72° ± 45°
Post-operative	149° ± 12°
Post-operative Nerot-Sirveaux classification	
No notching	3 (27%)
Grade 1	5 (45%)
Grade 2	1 (9%)
Grade 3	2 (18%)
Grade 4	0 (0%)
Post-operative VAS pain score	1.1 ± 1.4
Post-operative ASES score	80 ± 14

*VAS*, visual analog scale; *ASES*, American Shoulder and Elbow Surgeons.

Continuous variables are presented as average ± standard deviation and categorical variables as n (%).

∗ Statistically significant *P* ≥ .050.

Seven of 11 patients at a minimum of 2 years post-operatively had AFE measured at 40 ± 15 months (range: 24-58 months) post-operatively. Pre-operatively, patients had an AFE of 72° ± 45°, and post-operatively patients had an AFE of 149° ± 12° (*P* = .006) (Table II).

Post-operative VAS pain score was 1.1 ± 1.4, and ASES score was 80 ± 14 for the 11 patients we had greater than 2-year PROs (Table II). Eight of these patients also had a pre-operative VAS pain score (average, 4.1 ± 2.0) with an improvement of 3.3 ± 2.9 (*P* = .016) at the > 2-year follow-up, and pre-operative ASES score (average, 51 ± 16) with an improvement of 32 ± 25 (*P* = .008) at the > 2-year follow-up. At 25 months post-operatively, 1 patient was revised to an antibiotic spacer due to a recurrent infection with an rTSA implanted during a second stage 3 months after the antibiotic spacer. This patient received a third revision rTSA due to aseptic loosening 37 months post-operatively from the 2-stage revision rTSA and at 20 months post-operatively has a VAS pain score of 0 and an ASES score of 72.

## Discussion

This study evaluated the short-term to mid-term radiographic and functional outcomes of revision rTSA with allograft reconstruction for contained glenoid defects using a long-post baseplate with a 155° neck shaft angle humeral component. Overall, there was radiographic evidence of graft incorporation for 100% of patients at an average of 4 years post-operatively. Furthermore, 8 of the 11 patients (73%) had either Nerot-Sirveaux Grade 1 or no scapular notching. Additionally, AFE improved from 95.0° pre-operatively to 143.8° post-operatively. At the final follow-up, patients reported low pain with an average VAS pain score of 1.1 and very good functional scores with an average ASES score of 80.

In the setting of rTSA, prior reports of the use of an allograft for the treatment of glenoid defects vary with graft healing rates of 30%-86%.^9^^,^^11^^,^^14^^,^^16^^,^^19^ However, both primary and revision rTSA cases were included, and various types of glenoid defects were treated. For revision rTSA patients with severe uncontained defects treated using a large structural allograft, we have previously reported a failure of graft healing of 41.2% at a minimum of 5 years.^7^ We had previously published on shorter term outcomes in the uncontained defects in primary and revision rTSA treated with allograft reconstruction at a minimum of 2 years post-operatively and noted 82% complete healing.^20^ This healing rate in primary and revision rTSA for allograft reconstruction for uncontained defects lowered to 66.7% at a minimum of 5 years post-operatively.^7^ In the present study, only contained glenoid defects that were treated with an allograft during revision rTSA were included. All patients showed radiographic evidence of graft incorporation, although one patient was revised to an antibiotic spacer 25 months post-operatively due to a recurrent infection. The data support that contained and uncontained defects display distinct characteristics and therefore allograft may be best suited for contained as opposed to uncontained defects. In the future, classifying glenoid deformity accurately, using the contained/uncontained groupings or a more sophisticated 3-dimensional classification, in revision shoulder arthroplasty will be critical to accurately assess outcomes as baseline glenoid deformity likely influences both clinical and radiographic outcomes. Based on our prior study of uncontained defects, we will need to continue to monitor these patients to determine if there is an increase in failures over time as was identified in the uncontained cohort.

The patients in the present study had an average beta angle of 83.2° pre-operatively and 89.1° at the final follow-up supporting that we were able to accurately restore the inclination to at least neutral inclination. Residual superior baseplate tilt should be avoided in these cases as this may lead to bone graft failure. Additionally, 3 patients (27%) had no scapular notching and 5 patients (45%) had slight scapular notching. These data are consistent with prior published data on scapular notching after a Grammont style implant.^12^ While a more lateralized glenoid construct and a more varus inclination angle on the humeral implant may reduce notching, the goals of treatment in these severe cases were to maximize the chances of graft healing and lower the risks of instability; therefore, a medial glenoid/valgus humeral inclination design was used. Furthermore, patients' AFE significantly improved from 95.0° pre-operatively to 143.8° post-operatively consistent with previously reported outcomes after rTSA with structural allografting.^19^ Finally, patients reported an average VAS pain score of 1.1 and ASES score of 80, indicative of low pain and very good function. We have previously reported the clinical outcomes after revision rTSA using a Grammont-style implant and noted an average VAS pain score of 2.4 and ASES score of 61.^20^ Lee et al previously reported the clinical outcomes after a Grammont-style implant in the primary setting and at 2 years reported VAS pain score of 1.7 and ASES score of 78.^10^ The improved results in the current series compared to the prior revision cohort are likely due to most patients in the current series had loose glenoid and once treated with an allograft reconstruction, the patients clinically achieved the results more comparable to a primary rTSA than a revision because the humeral anatomy and posterosuperior cuff was in general maintained.

Of the original cohort of 16 patients, 4 developed a post-operative infection requiring revision. One occurred at 25 months post-operatively, while the others occurred prior to 24 months. However, the patient with an infection at 25 months post-operatively was a smoker at the time of surgery and subsequent revision to an antibiotic spacer, but quit prior to second-stage revision rTSA implantation. Smoking increases the risk for infections post-operatively^1^ and may have played a role in the patient's failed revision. In all cases of revisions in this series, they had a negative workup for infection including sedimentation rate, C-reactive protein, and aspiration. None of the cases were for a failed implant that had a prior infection or spacer. No cultures were taken at the time of revision which is standard protocol in aseptic loosening cases or rotator cuff insufficiency cases unless pre-operative laboratory examinations support a potential infection. Consequently, the data support that even in the setting of a loose implant or rotator cuff tear, there is not an inconsequently chance for infection (25% in this series) in the post-operative setting and patients and surgeons need to be aware of this risk. Nevertheless, if infection can be avoided, graft healing is predictable along with restoration of function and pain relief at short to mid term.

This study has limitations. First, this was a retrospective study design of only a small series of patients from a single surgeon. However, this is the first study to report on the radiographic and functional outcome of patients who underwent revision rTSA with allograft reconstruction specifically for contained glenoid defects using the described technique. Only one implant design was primarily used (Grammont-style implant with a long-post baseplate without the addition of biologics); therefore, translation of the data to other baseplate constructs or reconstructions using biologics must be done with caution. Additionally, healing and other radiographic measurements were made on plain radiographs rather than CT scans and may not be as accurate as those in prior studies using CT scans. Finally, only a minimum 2-year follow-up was obtained with an average follow-up of 4 years. Prior studies have shown bone grafts can fail in the intermediate term (2-5 years post-operatively).^7^ Therefore, future studies should report the outcomes of these patients at a minimum of 5 years.

## Conclusion

In the short to mid term, revision rTSA with allograft reconstruction for contained central glenoid defects using a long-post baseplate provides excellent radiographic healing, improved range of motion, good function, low pain, and low baseplate revision rates. This technique may represent an effective option for managing contained central glenoid defects in the setting of revision rTSA.

## Disclaimers:

Funding: No funding was disclosed by the authors.

Conflicts of interest: Christopher D. Joyce reports that he is a paid consultant for Zimmer Biomet.

Peter N. Chalmers reports that he is a paid consultant for DePuy and Advita Ortho; is a paid speaker for DePuy; receives intellectual property royalties from DePuy, Responsive Arthroscopy, and Advita Ortho; has stock in TitinKM Biomedical and Cinch Biomedical; receives research support from DePuy and the National Institutes of Health; and serves on the editorial board of the Journal of Shoulder and Elbow Surgery Reviews, Reports, and Techniques.

Robert Z. Tashjian reports that he is a paid consultant for Stryker; has stock in Conextions and Shoulder Innovations and Genesis; receives intellectual property royalties from Zimmer Biomet, Stryker, and Shoulder Innovations; receives research support from Stryker and the Department of Veteran's Affairs; receives publishing royalties from Springer; and serves on the editorial board of the Journal of Shoulder and Elbow Surgery.

Any additional authors, their immediate families, and any research foundations with which they are affiliated have not received any financial payments or other benefits from any commercial entity related to the subject of this article.

## Supplementary Data

Contained Defects Glenoid Graft
